# Synchronous solid pseudopapillary neoplasm and invasive ductal carcinoma of the pancreas: a case report

**DOI:** 10.1186/s40792-020-00969-9

**Published:** 2020-08-07

**Authors:** Chikanori Tsutsumi, Toshiya Abe, Yusuke Sawatsubashi, Sadafumi Tamiya, Daisuke Kakihara, Kazuyoshi Nishihara, Toru Nakano

**Affiliations:** 1 Department of Surgery, Kitakyushu Municipal Medical Center, 2-1-1 Bashaku, Kokurakita-Ku, Kitakyushu, 802-0077 Japan; 2 Department of Surgery 1, School of Medicine, University of Occupational and Environmental Health, Kitakyushu, Japan; 3 Department of Pathology, Kitakyushu Municipal Medical Center, Kitakyushu, Japan; 4 Department of Radiology, Kitakyushu Municipal Medical Center, Kitakyushu, Japan

**Keywords:** Solid pseudopapillary neoplasm, Invasive ductal carcinoma, Pancreatic neoplasm, Synchronous, Pancreatitis

## Abstract

**Background:**

Solid pseudopapillary neoplasm (SPN) of the pancreas is an extremely rare neoplasm with a favorable prognosis. On the other hand, pancreatic invasive ductal carcinoma (IDC) is known to be an aggressive malignancy. To the best of our knowledge, there is no report of SPN combined with IDC of the pancreas.

**Case presentation:**

A 66-year-old woman presented with abnormal genital bleeding and was diagnosed with inoperable cervical cancer. During computed tomography for cancer staging, the patient was incidentally diagnosed with pancreatic cancer. After radiation therapy for the cervical cancer, distal pancreatectomy with D2 lymph node dissection was performed. A postoperative pathological examination revealed SPN with ossification and well-differentiated IDC in the pancreatic body. On immunohistochemical staining, SPN tumor cells showed positive β-catenin and CD10 staining, whereas IDC cells were negative for both. The tumor boundaries were clear. Accordingly, the final pathological diagnosis was synchronous SPN and IDC of the pancreas. Moreover, pathological findings such as the ossification and small number of SPN cells suggested that SPN may have existed long before IDC initiation.

**Conclusions:**

Here, we report the first case of SPN combined with IDC of the pancreas. They may occur independently, and the long-term presence of SPN may lead to the development of IDC.

## Background

Solid pseudopapillary neoplasm (SPN) of the pancreas is extremely rare and shows favorable prognosis [1, 2]. In contrast, pancreatic invasive ductal carcinoma (IDC) is one of the most aggressive malignancies [3]. As far as we know, there are no reports on the relationship between SPN and IDC of the pancreas. Here, we present the first case of SPN combined with IDC of the pancreas and discuss the development of SPN and IDC of the pancreas.

## Case presentation

**Fig. 1:**
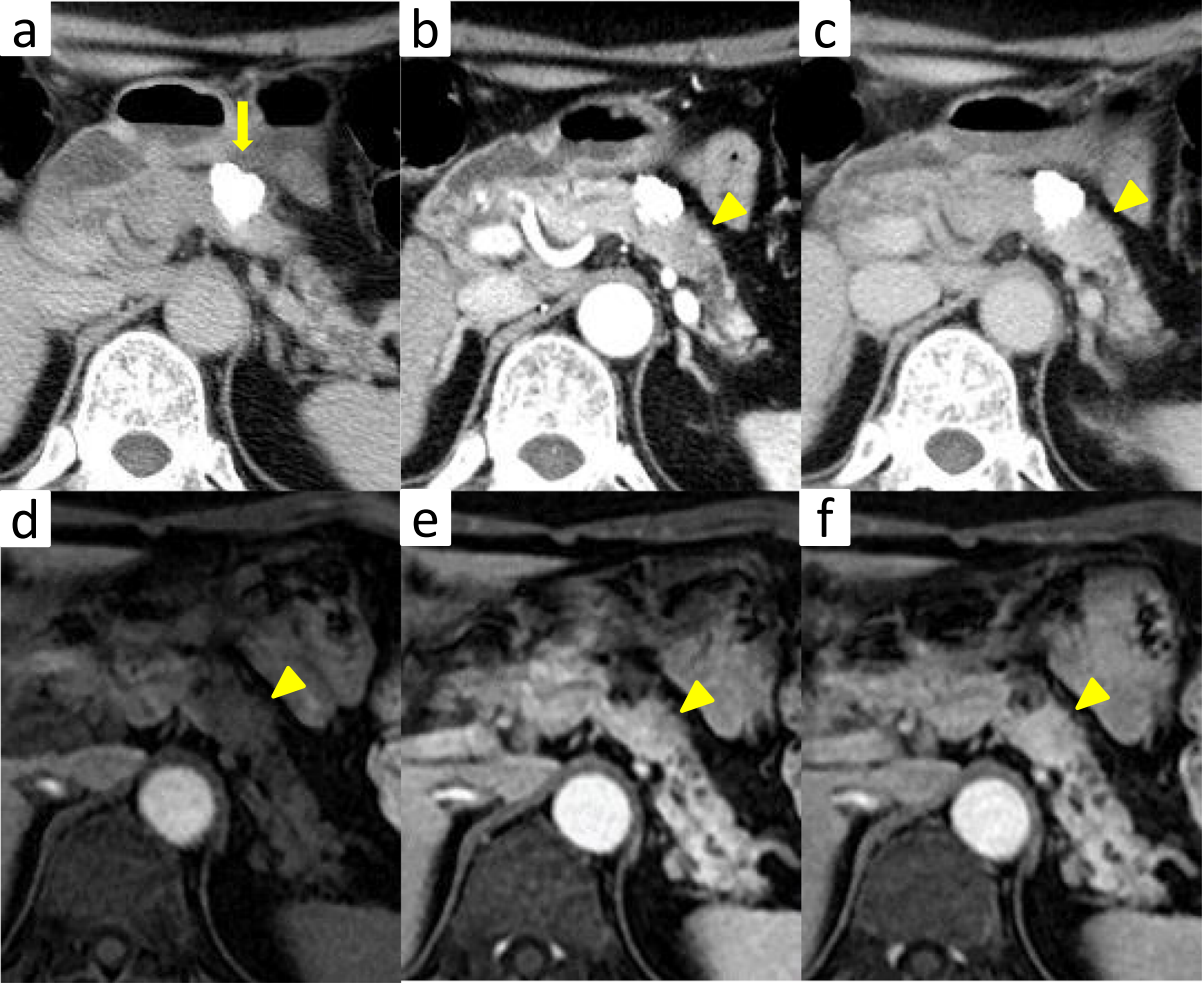
Plain CT, contrast-enhanced CT, and Gd-EOB-DPTA-enhanced MRI. Plain CT showing a calcification on the head side of the nodule (yellow arrowhead). Contrast-enhanced CT and Gd-EOB-DPTA-enhanced MRI showing a 21-mm nodule that showed gradually intense enhancement in the pancreatic body (yellow arrow). **a** Plain CT. **b** Arterial phase of CT. **c** Equilibrium phase. **d** Pre-enhancement phase of MRI. **e** Early phase of MRI. **f** Late phase of MRI. CT, computed tomography; Gd-EOB-DPTA, gadolinium-ethoxybenzyl-diethylenetriamine; MRI, magnetic resonance imaging

**Fig. 2:**
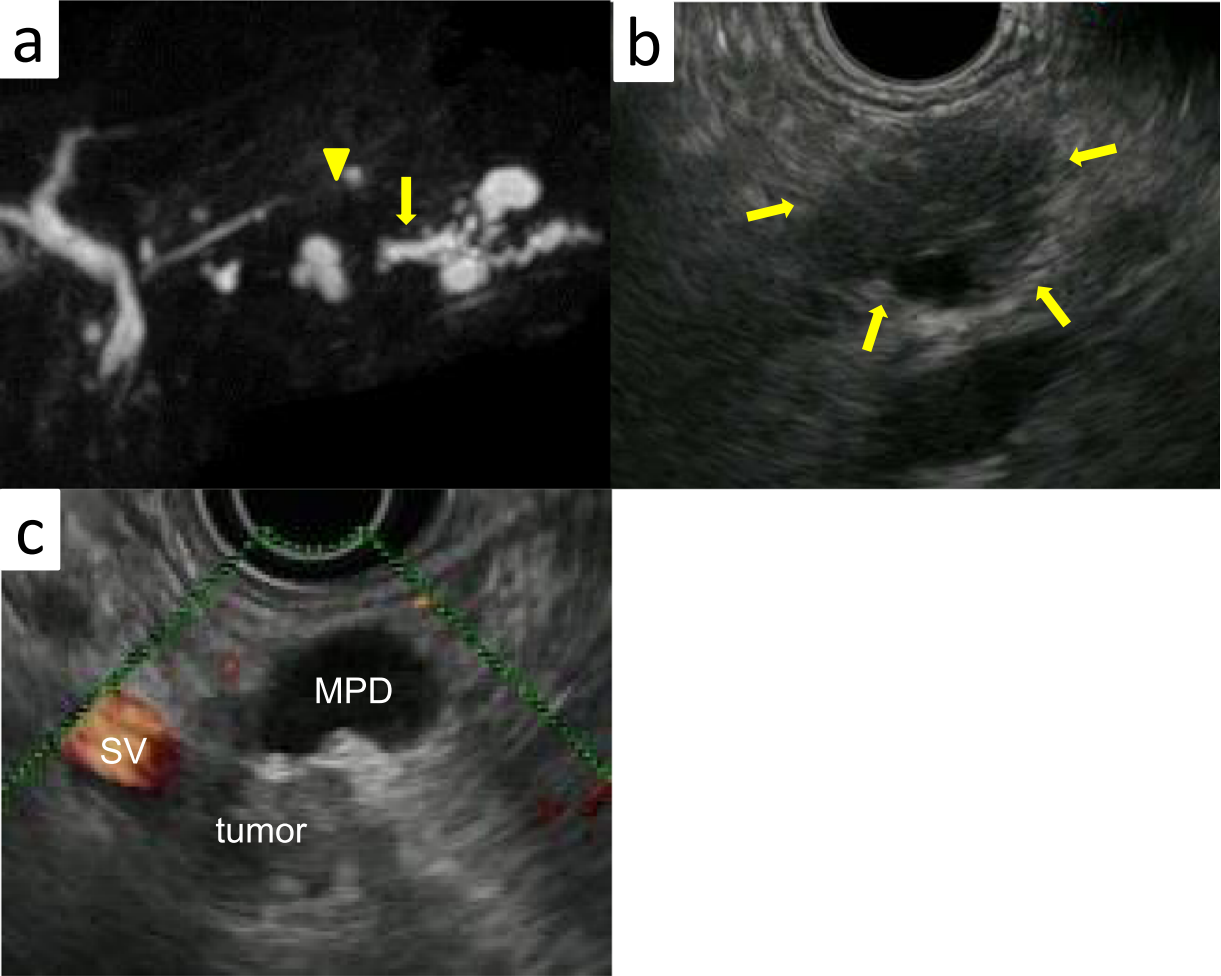
Magnetic resonance cholangiopancreatography examination. **a** Magnetic resonance cholangiopancreatography examination showing MPD disruption (yellow arrow) in pancreatic body and MPD dilation in the distal side of the disruption (yellow arrowhead). **b**, **c** EUS showed a 21-mm hypoechoic lesion near the calcification (yellow arrow). Invasion to the splenic vein by the tumor was suspected. MPD, main pancreatic duct; EUS, endoscopic ultrasonography; SV, splenic vein

**Fig. 3:**
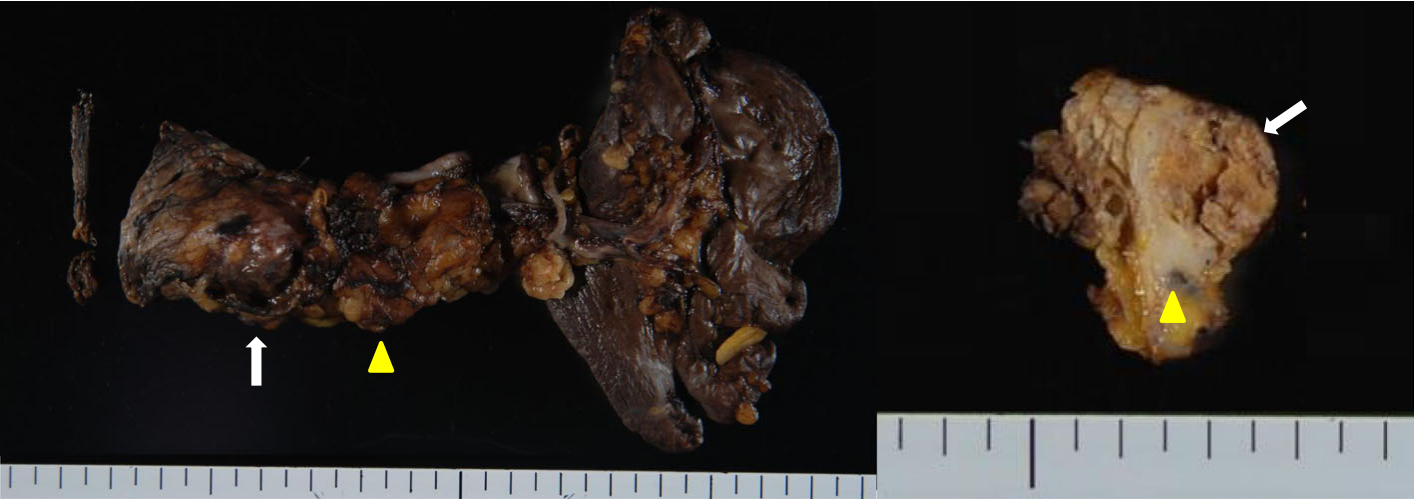
Macroscopic examination of the resected specimen. Macroscopic examination of the resected specimen showing a 20-mm hard mass (white arrow) and a 38-mm well-circumscribed nodule just distal to the mass (yellow arrowhead) in pancreatic body

**Fig. 4:**
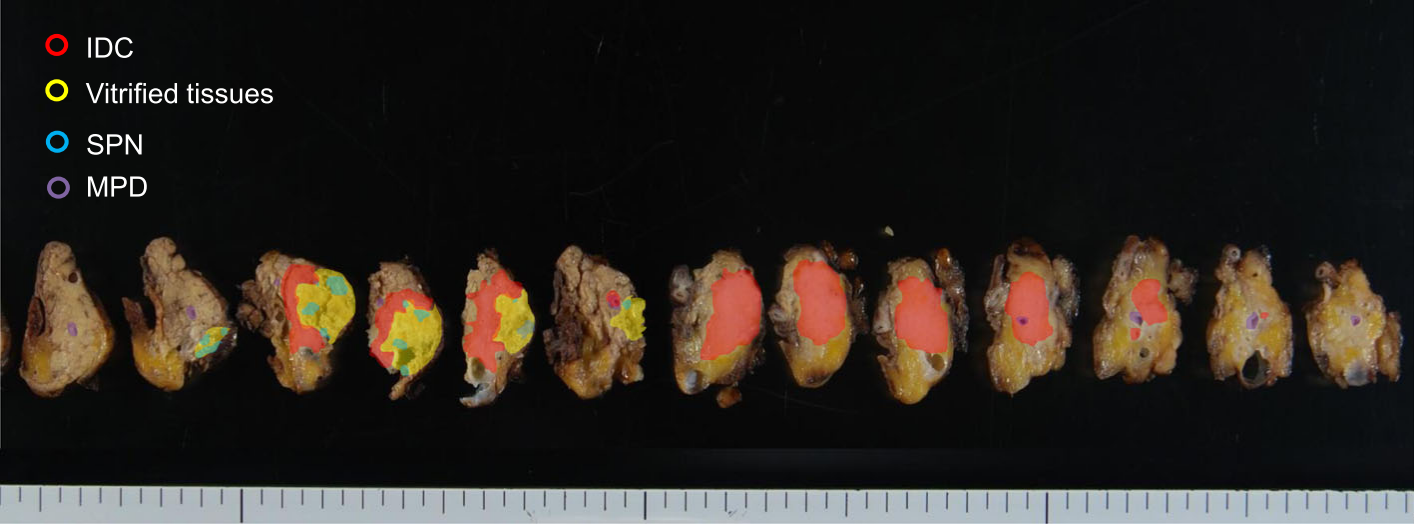
Tumor mapping showing IDC was adjacent to SPN in pancreatic body. IDC, invasive ductal carcinoma; SPN, solid pseudopapillary neoplasm

**Fig. 5:**
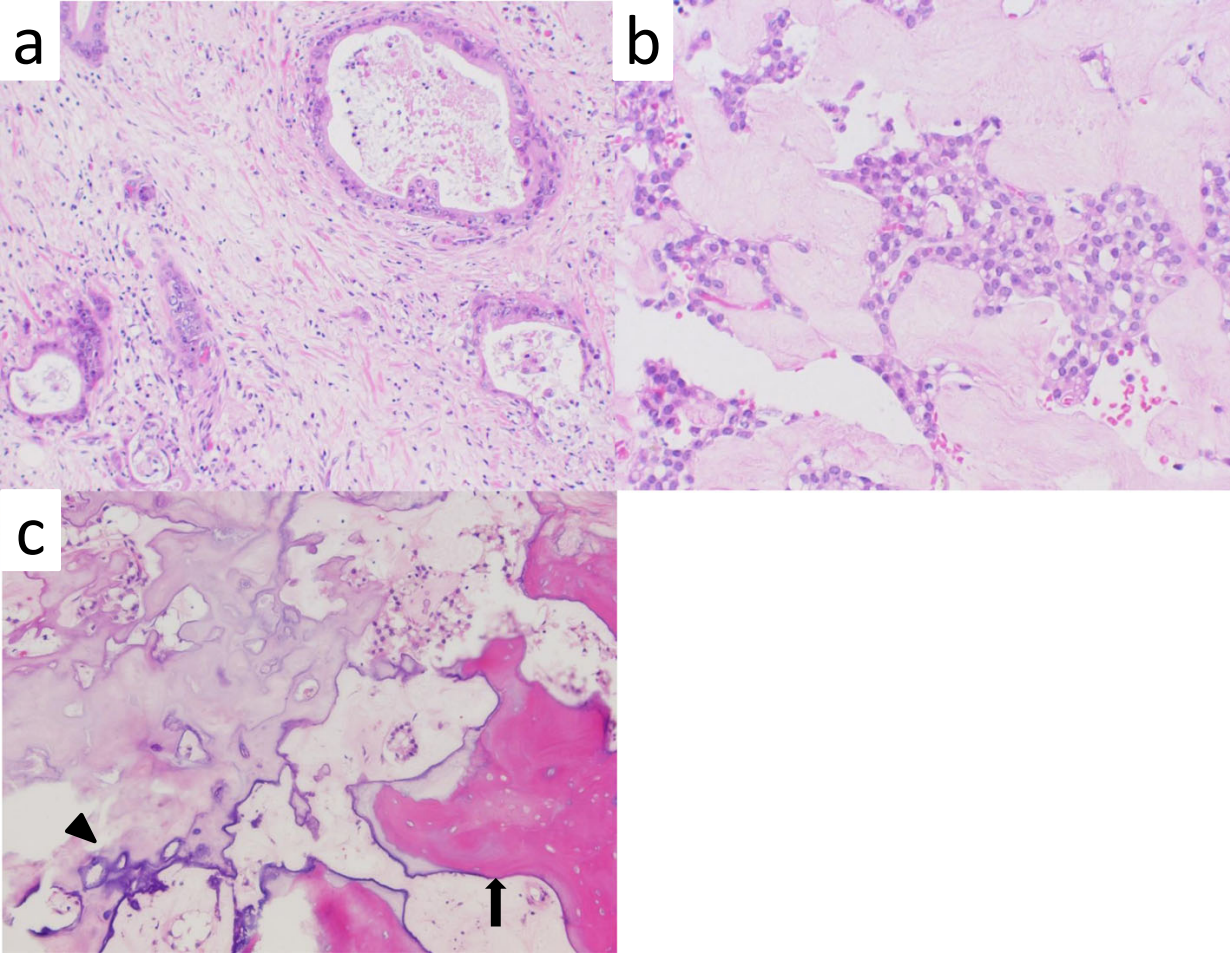
Histopathological findings (hematoxylin–eosin staining). **a** Well-differentiated invasive ductal adenocarcinoma (× 200 original magnification). **b** Vitrified tissues with areas containing uniform tumor cells (× 400 original magnification). **c** Calcification (black arrowhead) and ossification (black arrow) in the vitrified tissues (× 200 original magnification)

**Fig. 6:**
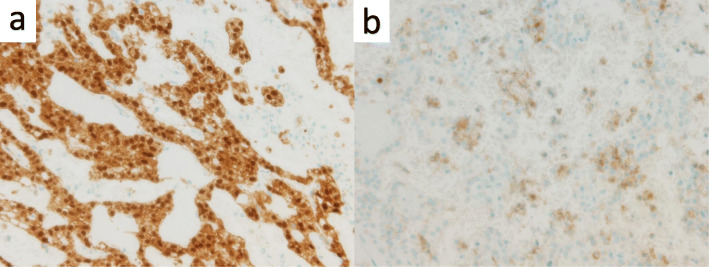
Histopathological findings (immunohistochemical staining). **a** Histopathological findings (immunohistochemical staining) showing positive staining for β-catenin and CD10. **a** β-catenin staining (× 400 original magnification). **b** CD10 staining (× 400 original magnification)

**Fig. 7:**
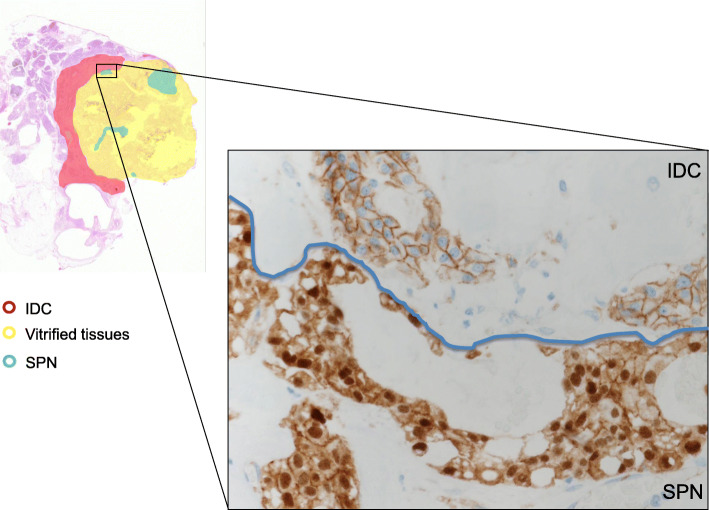
Tumor mapping and histopathological findings (immunohistochemical staining). The boundary between SPN (positive β-catenin staining) and IDC (no β-catenin staining) was clear (blue line)

A 66-year-old woman presented with abnormal genital bleeding. She was diagnosed with cervical cancer, and radical surgery could not be performed because of severe local invasion (stage IIIB; cT3bN1M0). Computed tomography (CT) for cancer staging incidentally revealed a pancreatic body tumor. The patient was referred to our hospital for further examination and treatment. After consulting with the gynecologist, we judged that it would take a long time to start treatment for cervical cancer if the pancreatic tumor was first examined closely and then resected. For this reason, the pancreatic tumor was inspected while performing radiation therapy for cervical cancer. She had no history of malignancies. Physical examination did not reveal any specific findings except for the abnormal genital bleeding. Tumor markers such as carcinoembryonic antigen (CEA) (5.1 ng/mL), carbohydrate antigen 19-9 (CA19-9) (749.8 U/mL), DUPAN-2 (320 U/mL), and Span-1 (69.0 U/mL) were elevated, while squamous cell carcinoma antigen and cancer antigen 125 were within normal range. Plain CT, contrast-enhanced CT, and gadolinium-ethoxybenzyl-diethylenetriamine pantaacetic acid (Gd-EOB-DPTA)-enhanced magnetic resonance imaging (MRI) revealed a 21-mm nodule in the pancreatic body that showed gradually intense enhancement, and a 20-mm calcification in the proximal side of the nodule (Fig. 1a–f). No enlarged lymph nodes or distant metastasis were evident. Magnetic resonance cholangiopancreatography showed main pancreatic duct (MPD) disruption and MPD dilation in the distal side of the disruption (Fig. 2a). Endoscopic retrograde cholangiopancreatography showed MPD disruption distal to the calcification. Endoscopic ultrasonography (EUS) demonstrated that there was a 21-mm hypoechoic lesion near the calcification, and invasion to the splenic vein was suspected (Fig. 2b, c). EUS-guided fine-needle aspiration cytology of the hypoechoic lesion revealed adenocarcinoma. Based on these findings, the patient was diagnosed with pancreatic cancer (stage IIA; cT3N0M0). The patient had been receiving radiotherapy for the cervical cancer for a month (external irradiation 50.4Gy/28Fr, intracavity irradiation 24Gy/4Fr) when distal pancreatectomy with D2 lymph node dissection was carried out. At the same time, splenectomy was also performed. The operating time was 236 min, and the blood loss volume was 60 mL. A macroscopic examination of the resected specimen showed a 20-mm hard mass in the pancreatic body and a 38-mm well-circumscribed nodule just distal to the mass (Figs. 3 and 4). A postoperative pathological examination revealed well-differentiated IDC in the nodule and confirmed perineural and anterior serosal invasion (Fig. 5a). There were no findings such as squamous cell components suggesting a relationship between pancreatic cancer and cervical cancer. In the hard mass, vitrified tissues with areas containing uniform tumor cells were found, presenting mild atypia, no lymphovascular invasion, and no infiltration to adjacent organs (Fig. 5b). In addition, calcification and ossification were also found in the vitrified tissues (Fig. 5c). Immunohistochemical staining revealed that uniform tumor cells from the hard mass were positive for β-catenin and CD10 (Fig. 6a, b), which were compatible with SPN. Additionally, the number of viable SPN cells was small, and the number of vitrified tissues was high. Furthermore, there was fibrosis with tissue destruction and exocrine parenchyma loss in pancreatic tissues distal to SPN. Coarse calcifications were thought to have been removed during cytohistologic preparation or eliminated by demineralization. The boundary between β-catenin-stained SPN cells and non-stained IDC cells was clear (Fig. 7). Accordingly, the final pathological diagnosis was SPN combined with IDC of the pancreas. The pancreatic IDC was classified as stage IIB (pT3N1M0) according to the 8th edition of the International Union Against Cancer Tumor–Node–Metastasis classification. Postoperatively, the patient followed an uneventful course and no complications. Three weeks after surgery, the patient received adjuvant chemotherapy with S-1, an oral fluoropyrimidine, at a dose of 100 mg/day (60 mg per body surface area). However, 4 weeks after surgery, she complained of strong backache, and CEA and CA19-9 were elevated to 8.3 ng/mL and 7088.6 U/mL, respectively. MRI revealed multiple vertebral metastases despite no bone metastasis preoperatively. Although chemotherapy sessions were not interrupted, she died 8 weeks after surgery.

## Discussion

SPN of the pancreas is a rare neoplasm, accounting for approximately 0.3 to 2.7% of all pancreatic tumors [1]. Most SPNs are diagnosed in young female patients under the age of 40 and reveal large tumors (mean tumor diameter 5.9 cm) [2]. These tumors are frequently located in the distal pancreas and are latent malignancies with excellent prognosis [2, 4]. Most patients are cured after radical surgery, with an increasing number of minimally invasive surgeries for SPN [5]. However, 10–15% of SPN cases have aggressive features, including lymphovascular invasion, adjacent organ invasion, and metastatic disease [6, 7]. Typical immunohistochemistry findings for SPN include positive staining for nuclear β-catenin and CD-10 [2, 8]. Characteristic imaging features of SPNs include a calcified peripheral rim. A previous study demonstrated calcification in 30.5% of SPN cases and ossification in only 3.3% [9]. The present case was a SPN with both calcification and ossification, who was older than the mean age of diagnosis. On the other hand, the prognosis of pancreatic IDC is extremely poor [3]. To the best of our knowledge, there is no literature of SPN combined with IDC of the pancreas, and the correlation with SPN and IDC remains to be clarified.

In the case presented here, SPN with no malignant features was adjacent to IDC. However, SPN was characterized by different grades of histologic atypia, with clear boundaries between them. Additionally, SPN was positive for β-catenin and CD10, but IDC was negative for these immunohistochemical stainings. As described above, we did not observe signs of histological transformation indicating a transition between SPN and IDC, and immunohistological features were different. These findings indicate that SPN and IDC may occur independently.

It is difficult to clarify what lesions preceded in this case using the preoperative assessment and the pathological findings. In the present case, the number of viable SPN cells was small, and the number of vitrified tissues was high. Additionally, ossification was also observed in the vitrified tissues. These findings suggest that SPN may have existed long before the development of IDC. Furthermore, the presence of SPN for a long period of time might have caused inflammation in the pancreatic tissue. Possible explanations for pancreatitis induced by SPN include two hypotheses. First, recurrent inflammation caused by SPN itself may affect surrounding pancreatic tissues. Iijima et al. [10] reported that calcification may be caused by recurrent inflammation and necrosis over a long period of time. In the present case, there was calcification without cystic components even inside the SPN. Furthermore, the patient was more than 20 years older than the mean age of diagnosis, which may suggest a more long-term presence of SPN. These findings suggest recurrent inflammation associated with SPN over a long period of time. Second, SPN with calcification and ossification could lead to MPD stenosis, resulting in inflammation of pancreatic tissues distal to SPN. Tajima et al. [11] reported a case of MPD stenosis caused by SPN with ossification. The present case presented SPN with calcification and ossification. Additionally, there was fibrosis with tissue destruction and exocrine parenchyma loss in pancreatic tissues distal to SPN, suggesting chronic pancreatitis. Based on these findings, SPN with calcification and ossification could lead to MPD stenosis, which might cause pancreatitis.

SPN cells show β-catenin staining in both the cytoplasm and nucleus due to the presence of a mutation in the gene that activates β-catenin expression. Aberrant cell cycle signaling through the β-catenin pathway has been associated with tumorigenesis in SPN and other cancers [12, 13]. Additionally, β-catenin signaling dysregulation is involved in chronic inflammation [14]. As discussed so far, pathological findings of the present case may support the correlation with long-term presence of SPN and chronic pancreatitis. Furthermore, chronic inflammation leads to the collapse of homeostatic interactions in the tissue microenvironment [14], and chronic pancreatitis is considered to be a high-risk factor for pancreatic cancer [15, 16]. Based on these findings, the long-term presence of SPN may contribute to the development of IDC, even though SPN is a low-grade malignant tumor. Further studies are needed to clarify the relationship between SPN and IDC.

In the present case, a prognostic factor was not cervical cancer but pancreatic cancer. However, the patient had not yet been diagnosed with pancreatic cancer at the time the patient was diagnosed with inoperable cervical cancer. Moreover, few studies had reported the efficiency of neoadjuvant chemotherapy for resectable pancreatic cancer at that time. For this reason, the pancreatic tumor was inspected while performing radiation therapy for cervical cancer, and subsequently, distal pancreatectomy was performed without neoadjuvant chemotherapy. The present case was diagnosed with resectable pancreatic cancer, which is currently a candidate for neoadjuvant chemotherapy [17]. Therefore, neoadjuvant chemotherapy and several watchful-wait interval for pancreatic cancer should be considered when the patient was diagnosed with pancreatic cancer and cervical cancer at the same time.

Postoperatively, the present case was diagnosed with multiple bone metastases, and she died 2 months after surgery. An autopsy was not performed because her family did not hope it, and the origin of bone metastasis remained unknown. Bone metastasis has been reported to occur in 5 to 20% of patients with pancreatic cancer, while in 1 to 16% of patients with cervical cancer [18–20]. Preoperatively, CEA, CA19-9, DUPAN-2, and Span-1 were elevated. However, tumor markers except for CEA and CA19-9 were not measured postoperatively. Furthermore, CEA and CA19-9 may be also elevated in cervical cancer. For this reason, it is difficult to predict a primary cancer from changes in tumor markers. Median survival of pancreatic cancer with bone metastasis was 7 months, while those of cervical cancer with bone metastasis was 34 months [21, 22]. Considering her course, the origin of metastasis may be pancreatic cancer rather than cervical cancer.

## Conclusions

We report the first case of SPN combined with IDC of the pancreas. The present case suggests that they may occur independently and the long-term presence of SPN may lead to the development of IDC.

## Data Availability

Not applicable.

## Abbreviations

SPN: Solid pseudopapillary neoplasm
IDC: Invasive ductal carcinoma
CEA: Carcinoembryonic antigen
CA19-9: Carbohydrate antigen 19-9
Gd-EOB-DPTA: Gadolinium-ethoxybenzyl-diethylenetriamine pantaacetic acid
MRI: Magnetic resonance imaging
MPD: Main pancreatic duct
EUS: Endoscopic ultrasonography
